# Endoscopic surveillance alone is feasible and safe in type I gastric neuroendocrine neoplasms less than 10 mm in diameter

**DOI:** 10.1007/s12020-022-03143-3

**Published:** 2022-07-27

**Authors:** Klaire Exarchou, Haiyi Hu, Nathan A. Stephens, Andrew R. Moore, Mark Kelly, Angela Lamarca, Wasat Mansoor, Richard Hubner, Mairéad G. McNamara, Howard Smart, Nathan R. Howes, Juan W. Valle, D. Mark Pritchard

**Affiliations:** 1 Department of Molecular and Clinical Cancer Medicine, Institute of Systems, Molecular and Integrative Biology, University of Liverpool, Liverpool, UK; 2 Department of Upper Gastrointestinal Surgery, Liverpool University Hospitals NHS Foundation Trust, Liverpool, UK; 3 Department of Gastroenterology, Beijing Friendship Hospital, Capital Medical University; National Clinical Research Center for Digestive Diseases, Beijing, China; 4 Department of Gastroenterology, Liverpool University Hospitals NHS Foundation Trust, Liverpool, UK; 5 Department of Gastroenterology, Manchester University NHS Foundation Trust, Manchester, UK; 6 Department of Oncology, The Christie NHS Foundation Trust, Manchester, UK; 7 Division of Cancer Sciences, University of Manchester, Oxford Rd, Manchester, UK

**Keywords:** Neuroendocrine tumour, Carcinoid, Stomach, Surveillance, Endoscopy, Surgery

## Abstract

**Purpose:**

Type I gastric neuroendocrine neoplasms (g-NENs) have a low risk of metastasis and a generally favourable prognosis. Patients with small type I g-NENs (≤10 mm) frequently require no treatment, whereas those with larger polyps usually undergo resection. We evaluated the safety and outcomes of endoscopic surveillance after no initial treatment in selected patients with type I g-NENs.

**Methods:**

Retrospective analysis of type I g-NEN patients across two European Neuroendocrine Tumour Society Centers of Excellence 2003–2019.

**Results:**

Following initial assessment, 87 of 115 patients with type I g-NEN (75 with polyps ≤10 mm) received no initial treatment and underwent endoscopic surveillance. 79/87 (91%) demonstrated no clinically meaningful change in tumour size or grade over a median 62 month follow up. Only two patients developed NEN progression that required a change in management and two other patients developed gastric adenocarcinoma/high grade dysplasia; all four initially had ≥11 mm g-NENs.

**Conclusions:**

Patients with ≤10 mm type I g-NENs were unlikely to develop clinically significant tumour progression and in most cases, resection was not needed. The endoscopic surveillance interval could therefore potentially be safely increased to every 2–3 years in such patients. However, lifelong surveillance is still advocated due to the additional risk of developing gastric adenocarcinoma.

## Introduction

Gastric neuroendocrine neoplasms (g-NENs) are relatively rare tumours and account for approximately 7% of all digestive neuroendocrine neoplasms (NENs) [1] and less than 1% of all gastric neoplasms [2]. However, their incidence has been increasing in most countries over recent decades. This is likely due to greater awareness of the disease among clinicians, improved diagnostic techniques and more widespread use of upper gastrointestinal endoscopy [1, 3]. g-NENs are subdivided into three main types [4, 5], each of which has a distinct biological behaviour, prognosis and management ([6] (Table 1). Type II g-NENs develop in the context of Zollinger-Ellison syndrome and multiple endocrine neoplasia type 1, whereas Type III g-NENs are sporadic lesions and are managed similarly to gastric adenocarcinoma [4].

**Table 1:** Types of gastric neuroendocrine tumours, general characteristics in endoscopic appearance, histology, and management

	Type I	Type II	Type III
**Proportion, %**	70–80	5–10	15–20
**Gastric localisation**	Corpus, fundus	Body, fundus, antrum	Antrum or corpus
**Typical endoscopic and morphological characteristics**	Single/multiple (60%), small (<10 mm); polypoid or submucosal	Often multiple, small (<10–20 mm); polypoid (sessile)	Single, large size (>20 mm); occasionally ulcerated
**Associated disorders**	Chronic atrophic gastritis and pernicious anaemia; achlorhydria	Gastrinoma/Multiple endocrine neoplasia 1 (MEN-1)	Sporadic
**Histology**	Well differentiated (G1-G2)	Well differentiated (G1-G2)	Well differentiated, poorly differentiated or mixed endo/exocrine (G1,2,3 NET or NEC)
**Fasting serum gastrin levels**	High	High	Normal
**Gastric acid**	Low	High	Normal
**Investigations**	• Endoscopic assessment: Number, size and location of tumour(s), tumour biopsies, assess background gastric mucosa, biopsies of gastric antrum and corpus, pH of gastric juice		
	• Biochemical assessment: Fasting plasma gastrin and chromogranin A, anti-gastric parietal cell and intrinsic factor antibodies, thyroid function tests, Full Blood Count, vitamin B12		
	• Histological assessment: Ki67% and mitotic index, Lymphovascular invasion grade. Gastric corpus: Atrophic gastritis, intestinal metaplasia, Enterochromaffin-like cell hyperplasia. Antrum: Gastrin cell hyperplasia and H. pylori infection		
	• Endoscopic ultrasound scan (EUS)		
	• CT/MRI scan		
	• Somatostatin Receptor Imaging		
**Management**	Tumours <10 mm	Treatment of gastrinoma and MEN-1	Partial or Total gastrectomy with LN dissection
	Endoscopic surveillance every 1–2 years		
			Systemic therapy for metastatic disease (chemotherapy, Somatostatin analogues, Peptide Receptor Nucleotide therapy)
	Tumours >10 mm		
	No Lymph node (LN) involvement and confined to submucosa/lamina propria -		
	Endoscopic resection		
	LN involvement and/or positive margin on endoscopic resection -		
	Surgery (wedge resection, subtotal/total gastrectomy)		
**Risk of metastases, %**	2–5	10–30	50–100
**Prognosis**	Excellent	Very good	Poor

Adapted from Current Oncology Rep [6]

Type I g-NENs, the focus of this paper, are by far the most common type and account for approximately 80% of cases. They are associated with autoimmune atrophic gastritis and hypochlorhydria. This gastric pathology results in elevated fasting serum gastrin concentrations (hypergastrinaemia), and this is thought to be the main factor that drives type I g-NEN development. Type I g-NENs are often small (the majority are <10 mm in diameter), multiple, located in the body/fundus of the stomach and low grade (grade 1 or low grade 2). The risk of metastatic potential is low and directly correlated with tumour size [7, 8]. A recent systematic review of type I g-NENs confirmed the indolent course of this tumour type with a very low disease-specific mortality [5]. Only five tumour-related deaths were reported in more than one thousand patients and all these patients had unusual disease characteristics, such as very large tumour size, grade 3 histology or metastatic disease at presentation. The current European Neuroendocrine Tumour Society (ENETS) guidelines therefore suggest that a conservative management approach is preferred over surgery in the majority of cases [9]. Conservative management for patients with polyps <10 mm in diameter usually entails annual or two-yearly endoscopic surveillance with biopsy sampling of polyps. Endoscopic resection is advised for lesions over 10 mm in diameter (where clinically appropriate and safe in view of patient-related factors). Conversely, some authors advocate resection of all visible lesions greater than 5 mm [10, 11]. Currently there are no randomised data to inform our practice and there are few studies comparing the outcomes of an aggressive endoscopic resectional approach (i.e., resection of all visible lesions) versus a more selective resectional strategy in only larger polyps, [7].

Modern management pathways for patients with type I g-NENs are burdensome for both patients and hospital endoscopy resources, as even those individuals who have an endoscopic resection still need to have lifelong endoscopic surveillance due to the multifocal nature of this tumour type. We therefore set out to review the outcomes of our longstanding endoscopy surveillance programmes for type I g-NENs at two ENETS Centres of Excellence in the Northwest of England, to establish how frequently patients showed evidence of tumour progression and whether this subsequently affected patient management or survival.

## Materials and methods

### Patient cohort

Details from institutional electronic records of all consecutive patients referred with type I g-NENs across two ENETS Centres of Excellence (Liverpool and Manchester, United Kingdom (UK)) between 2003–2019 were retrieved from a prospectively collected database. We performed a retrospective analysis of this, supplemented with data from institutional electronic patient records. Inclusion criteria were histologically confirmed type I g-NEN, details available about any interventional procedure performed, regular endoscopic follow-up conducted at a NEN Unit, or a Gastroenterology department affiliated with a NEN Unit. The project was registered and approved by the respective hospital Audit departments. Data collected, when available, included patient demographics, tumour characteristics (size, number, and location), biochemical and histological results, treatment plan and follow-up. After a baseline endoscopic assessment, all cases were discussed, and a management plan agreed at the institution’s NEN multidisciplinary team meeting.

### Patient assessment

Patients underwent a baseline endoscopic assessment after referral to the ENETS Centre of Excellence, which included detailed recording of tumour size, number, and site of lesions as well as evaluation for atrophic gastritis. Histological evaluation included determination of tumour grade, Ki67 index, and assessment for the presence of atrophic gastritis, intestinal metaplasia, and enterochromaffin-like cell (ECL) hyperplasia in background antral and corpus biopsies. For some patients managed at the start of the programme, Ki67 immunohistochemistry was not routinely performed, hence these results were not all available. All the patients were endoscoped by experienced NEN gastroenterologists (DMP, ARM, MK), which reduced interobserver variability. Polyps were assessed by white light endoscopy, assisted by narrowband imaging, if clinically indicated. Tumour size was estimated endoscopically by comparison with an opened pair of biopsy forceps (Single-Use Radial Jaw 4; Boston Scientific, Hemel Hempstead, UK).

Evidence of concurrent or past *H. pylori* infection was determined by gastric histology and/or a rapid urease test and (at one of the sites only) by serology testing. Blood tests performed were full blood count, vitamin B12 levels, iron studies, thyroid function tests, anti-gastric parietal cell and anti-intrinsic factor antibodies, as well as a blood fasting gut hormone profile (including measurement of gastrin and chromogranin A concentrations).

### Patient management and endoscopic surveillance algorithm

A few patients underwent endoscopic polypectomy at their referring general hospital prior to referral to the ENETS Centre of Excellence. After referral, patients underwent a baseline endoscopic assessment, as described above. In general, it was recommended that patients who were found to have small polyps (≤10 mm) were enrolled on an endoscopic surveillance programme, with the intention of delivering no specific treatment unless there was a change in tumour characteristics. Patients who had polyps 11–20 mm diameter were counselled about the relatively indolent nature of these tumours and were offered endoscopic resection (if safe and feasible) and if they declined or resection was considered high risk, endoscopic surveillance. Many patients in this category who were elderly or who had significant comorbidities therefore elected to have endoscopic surveillance, particularly if they had multiple polyps which would render endoscopic resection more hazardous. Patients who had polyps ≥21 mm in diameter were considered for surgery.

Patients were considered for intervention if they had a clinically meaningful change in NEN size or grade or developed a different type of malignancy in the upper gastrointestinal tract. For g-NENs that initially measured ≤10 mm, the trigger for such an evaluation was an increase in size to greater than 15 mm, for polyps that initially measured >11 mm, the trigger was an increase in size of at least a further 5 mm and the trigger for an increase in grade was from G1 to G2. In the early 2000s there was also a vogue to perform antrectomy on patients who had multiple type I g-NENs to remove the anatomical source of hypergastrinaemia, so a few patients with small tumours opted for this treatment at that time (as previously described in [12]). Furthermore, during our study period, eight patients also participated in an open label phase II trial (NCT01339169) to assess the efficacy, tolerability, and safety of netazepide, a gastrin receptor antagonist. Netazepide was shown to decrease the size and number of polyps present and these effects were reversed off treatment [13]. Additional endoscopies that were performed as a part of the trial protocol have been removed from our analyses.

All patients who were offered or chose to have no treatment or who underwent initial endoscopic resection were subsequently enrolled into an endoscopic surveillance programme. The first endoscopic surveillance was scheduled six-twelve months after diagnosis or endoscopic resection. If no further changes were confirmed on endoscopy (number and size of tumours) and histology (grade and Ki67), annual or biennial subsequent endoscopies were performed (usually) by the same endoscopist. Any persistent lesions seen during follow-up endoscopy were biopsied on each occasion. Biopsies from the background mucosa were also assessed histologically if any concerning features were observed.

### Statistical analysis

Descriptive variables are summarised as medians with interquartile range (IQR). A Kruskal-Wallis test was used for non-normally distributed data for comparisons between treatment groups. Overall survival, intervention free survival and rate of change were calculated using the Kaplan–Meier method and compared using the log-rank test. Differences were considered significant at *p* < 0.05. Statistical analysis was performed using GraphPad Prism version 8.4.0 for Windows, GraphPad Software, La Jolla California USA, www.graphpad.com.

## Results

### Cohort characteristics

115 patients were identified with type I g-NENs over a 16-year period. Their initial clinical characteristics are described in Table 2. Median age at the time of referral for the whole cohort was 64 years (IQR:50–73) with a small female gender predominance (60%). The overall median follow-up was 66 months (IQR:38–113). Anti-parietal cell and intrinsic factor antibodies were present in 87% and 17% of patients who were tested. All patients demonstrated chronic atrophic gastritis affecting the corpus and 84/115 (73%) also had histological evidence of intestinal metaplasia. Seven patients out of 115 (6%) had histological evidence of current *H. pylori* infection at index endoscopy and 9/86 (10%) patients who had no current evidence of *H. pylori* infection by histology had positive IgG antibodies by serology. 62/91 (68%) patients had evidence of vitamin B12 deficiency either prior to NEN diagnosis or on baseline measurement of haematinics after referral. 38% of patients had evidence of hypothyroidism (as an example of another autoimmune disease) at the time of referral or were already receiving thyroxine treatment for hypothyroidism.

**Table 2:** Baseline characteristic of Type I g-NEN patients

Patient characteristics (*n* = 115)	
**Age at diagnosis (years)**	64 (IQR:50–73)
**Female gender,** ***n***	69 (60%)
**Proton pump inhibitor use,** ***n*** **(%)**	47/115 (41%)
***H. pylori*** **serology positive,** ***n***	9/86 (10%)
***H. pylori*** **histology positive,** ***n***	7/115 (6%)
**Positive anti-parietal cell antibodies,** ***n***	76/87 (87%)
**Positive anti-intrinsic factor antibodies,** ***n***	16/88 (18%)
**Gastrin, (*N*** < 40 **pmol/L),** ***n*** **(%)**	
**40–100**	2 (2%)
**100–400**	34 (40%)
**>400**	50 (58%)
**Chromogranin A, (*N*** < **150pmol/L) (*n*** = **80)**	33 (IQR: 10–43)
**Haemoglobin, (*N*** **130–160** **g/L) (*n*** = **91)**	127 (IQR107–140)
**Ferritin, (*N*** **30–400** **ug/L) (*n*** = **88)**	25 (IQR: 11.5–63.5)
**Folate, (*N*** **4.6–18.7** **ug/L) (*n*** = **88)**	8.2 (IQR:6.2–10.2)
**Hypothyroidism,** ***n***	33/88 (38%)
**Vitamin B12 deficiency (*N*** **191–663** **ng/L),** ***n***	62/91 (68%)
**Chronic atrophic gastritis on corpus biopsy,** ***n***	115 (100%)
**Intestinal metaplasia on gastric biopsy,** ***n***	84 /115 (72%)
**Initial treatment plan,** ***n***	
**Endoscopic Surveillance**	87 (76%)
**Endoscopic Resection**	10 (9%)
**Surgery**	8 (7%)
**Excluded from study**	10 (9%)
**Median follow up, months**	
**All**	66 (IQR: 38–113)
**Endoscopic Surveillance**	62 (IQR: 37–114)
**Endoscopic Resection**	70 (IQR: 64–112)
**Surgery**	87 (IQR: 24–110)

### Clinicopathological features

The majority of patients (100/115) had multiple gastric polyps identified during their baseline endoscopy (Table 3). Almost all the polyps, were located in the gastric body/fundus. There was no difference in the total number of gastric polyps between the different treatment groups. Overall, the median size of the largest individual polyp was 6 mm (IQR: 5–10 mm). Patients in the endoscopic surveillance group had smaller maximum polyp size (6 mm) when compared to patients in the endoscopic or surgical resection groups (12 mm, *p* = 0.001 and 13 mm, *p* = 0.01 respectively). Furthermore, patients in the endoscopic resection group had a higher Ki67 index (median = 2, IQR:2–9) when compared to patients in the surveillance group (median = 1.5, IQR:1–2, *p* = 0.003). No grade 3 (G3) NENs or poorly differentiated neuroendocrine carcinomas (NECs) were identified in this cohort at baseline.

**Table 3:** Baseline oesophagogastroduodenoscopy (OGD) and histology findings

Baseline OGD and Histology Findings						
	Overall	No follow up	Surveillance	Endoscopic treatment	Surgery	
**Number of polyps,** ***n***						
**1**	15 (13%)	2 (20%)	12 (14%)	1 (10%)	0	
**2–10**	64 (56%)	6 (60%)	48 (55%)	5 (50%)	5 (63%)	
**10–20**	15 (13%)	1 (10%)	12 (14%)	1 (10%)	1 (13%)	
**>20**	21 (18%)	1 (10%)	15 (17%)	3 (30%)	2 (25%)	
**Tumour size, mm**	6	6	6	12	13	
	(IQR:5–10)	(IQR:3–14)	(IQR:4–10)	(IQR:10–13)	(IQR:7–30)	
**Tumour size (mm),** ***n***						Surveillance vs ER 0.0015
**≤10**	92 (80%)	7 (70%)	75 (86%)	4 (40%)	2 (25%)	Surveillance vs Surgery 0.0142
**11–20**	19 (16%)	2 (20%)	12 (14%)	6 (60%)	3 (38%)	Kruskal-Wallis
**21–30**	1 (1%)	1 (10%)	0	0	0	
**>30**	3 (3%)	0	0	0	3 (38%)	
**Tumour site,** ***n***						
**Cardia**	2 (2%)	1 (10%)	1 (1%)	0	0	
**Body/Fundus**	107 (93%)	7 (72%)	82 (94%)	10 (100%)	8 (100%)	
**Antrum**	1 (1%)	0	1 (1%)	0	0	
**Unknown**	5(4%)	2 (20%)	3 (3%)	0	0	
**Grade,** ***n*** **(%)**						
**G1**	76 (66%)	8 (80%)	60 (69%)	6 (60%)	2 (25%)	
**G2**	18 (16%)	1 (10%)	12 (14%)	4 (40%)	1 (13%)	
**Unknown**	21 (18%)	1 (10%)	15 (17%)	0	5 (63%)	
**Ki67%**	2	1	1.5	2	2	
	(IQR:1–2)	(IQR:1–2)	(IQR:1–2)	(IQR:2–9)	(IQR:1–3)	

### Patient management

**Fig. 1:**
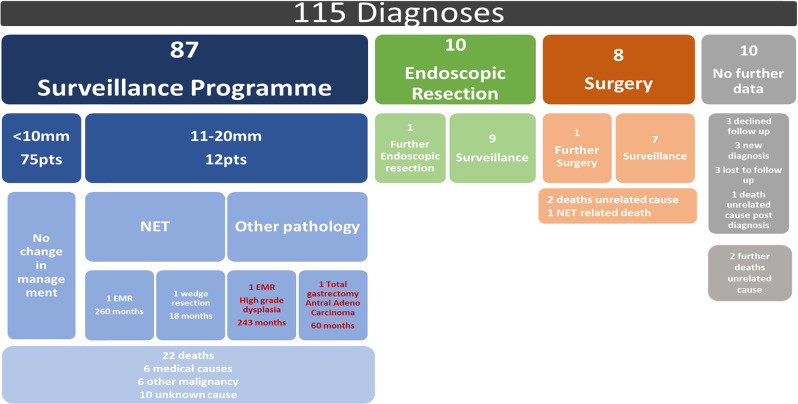
Flowchart of Type I g-NEN patient cohort according to management

There were no follow-up data available for ten patients due to them having a recent diagnosis (*n* = 3), being lost to (*n* = 3) or declining (*n* = 3) follow-up or dying from an unrelated cause shortly after referral (*n* = 1). These patients were therefore removed from further analyses. The remaining 105 patients were then divided into three groups based on their initial management plan. Ten patients initially underwent an endoscopic resection, and eight patients initially had surgery (7 antrectomy, 1 total gastrectomy). The remaining 87 patients received no initial treatment and were enrolled into an endoscopic surveillance programme, as outlined in Fig. 1.

### Endoscopic resection group

Ten patients underwent endoscopic resection as primary therapy, either just prior to or immediately after referral to the ENETS Centre of Excellence. These NENs were larger in size (median 12 mm, IQR:10–13 mm) and four had grade 2 (G2) pathology. No perioperative complications were reported. Only one patient progressed to require another endoscopic resection of a new 8 mm G2 NEN at a different site in the stomach, 35 months after diagnosis and 14 months after the first endoscopic resection. All patients are still alive and remain on endoscopic surveillance, with no further changes being found and have had a median of 3 further follow-up endoscopies/patient.

### Surgery group

Eight patients underwent surgical resection, and all these procedures were performed prior to 2011. Seven patients underwent an antrectomy to remove the anatomical source of hypergastrinaemia. One of these patients had a subsequent completion subtotal gastrectomy for a persistent 15 mm G2 NEN, 48 months after their first procedure (as previously described in [12]). All patients received regular annual post-operative endoscopic surveillance. Six of the seven patients were subsequently discharged, as their NENs completely regressed, but two of them have subsequently died of unrelated conditions. One patient initially underwent a total gastrectomy for a 50 mm grade 1 (G1) type 1 g-NEN with lymph node metastasis being found at diagnosis; the patient died 3 years post operatively from metastatic NEN and that is the only known tumour-related death in the whole cohort.

### No initial treatment and endoscopic surveillance group

87 patients had no initial treatment and were enrolled into a periodic endoscopic surveillance programme. 75 of these patients (86%) had multiple small NENs ≤10 mm in diameter. 69% of the tumours were G1, with a median Ki67 index of 1% (IQR:1–2%) (Table 3). The grade was unknown in 17%. 79 (91%) of these 87 patients did not demonstrate any change (as defined in Methods) in the number, size, or grade of tumours during follow up. During follow-up, 445 endoscopies were performed, with a median of 4 per patient (IQR:2–6). Median time to first surveillance endoscopy was eight months (IQR:3.5–13 months) and median time between endoscopies was 14 months (IQR:12–20). Overall, the median patient follow-up was 62 months (IQR:37–114). 22 deaths have been recorded in this group, but there were no known NEN-related deaths (six died from other medical conditions, six died from other malignancies and 10 causes of death are unknown, as they resided at a distance from the ENETS Centers of Excellence and the management of their final condition did not occur at our hospitals).

### Tumour Progression in the endoscopic surveillance group

Only six patients who initially had no treatment and who were enrolled into the surveillance programme demonstrated a change (as defined in Methods section) in NEN size or grade during follow up (Table 4). Two other patients developed other gastric malignancies during follow up. These outcomes were analysed according to baseline tumour size to determine the extent to which this influenced outcomes:

**Table 4:** Changes identified during follow up period (EMR, endoscopic mucosal resection; OGD, oesophagogastroduodenoscopy)

Changes during follow up period									
Patient number	Age at diagnosis	Sex	Initial treatment plan	Baseline OGD findings			Change	Time interval (months)	Management Plan
				Size (mm)	Grade	Polyp Number			
**1**	40	Female	EMR	10	2	1	New 8 mm, Grade 2 polyp	35	EMR
**2**	75	Male	SURGERY	15	2	1	Persistent 15 mm polyp after antrectomy	28	SURGERY: Subtotal gastrectomy
**3**	38	Female	SURVEILLANCE	5	1	10–20	Increase in Grade (Ki67 2–4.8%)	87	SURVEILLANCE: Repeat OGD, Ki67 2%
**4**	51	Male	SURVEILLANCE	6	1	2–10	Increase in Grade (Ki67 2–5%)	50	SURVEILLANCE: Repeat OGD, Ki67 2%
**5**	70	Female	SURVEILLANCE	10	1	10–20	Increase in size (10–20 mm)	50	SURVEILLANCE: patient choice
**6**	65	Female	SURVEILLANCE	13	1	2–10	Increase in size (1–20 mm)	260	EMR: Histology tiny focus of G3 NET, Ki67 40%, nil on follow-up
**7**	74	Male	SURVEILLANCE	20	1	2–10	Increase in Grade (Ki67 2–20%)	7	SURVEILLANCE: Repeat OGD, Ki67 5%
**8**	81	Female	SURVEILLANCE	20	1	2–10	Increase in size (20–30 mm)	18	SURGERY: Wedge resection, G1
**9**	76	Male	SURVEILLANCE	15	1	>20	Antral adenocarcinoma	60	SURGERY: Total gastrectomy for pT1bN1 R0, later died from cholangiocarcinoma
**10**	73	Male	SURVEILLANCE	12	1	2–10	High grade dysplasia	243	EMR: High grade dysplasia, nil on follow-up

#### ≤10 mm tumours at baseline (75 patients)

No patients demonstrated a clinically meaningful change in tumour size. Three patients however developed an increase in tumour grade from 1 to 2. All these patients however had multiple co-morbidities, precluding endoscopic therapy or surgery, therefore endoscopic surveillance was continued. During further follow-up, two patients were downgraded to G1, whilst one patient has had a persistent G2 (Ki67 5%) polyp.

#### 11–20 mm tumours at baseline (12 patients)

Three patients had an increase in polyp size greater than 5 mm from their baseline endoscopy. One patient had increase in size from 11 to 20 mm of a G1 (Ki67 1%) polyp after 50 months follow-up. A ^68^Ga DOTATOC PET/CT scan demonstrated a tracer avid lymph node adjacent to the lesser curvature of the stomach, but no additional somatostatin receptor positive pathology. This lymph node was present on the Computed Tomography (CT) scan at diagnosis and has minimally changed in size over 50 months. Given the indolent course of this tumour and after careful counselling, the patient opted to have ongoing surveillance management.

The second patient underwent an endoscopic mucosal resection 260 months after initial diagnosis for an ulcerated 20 mm NEN. Her baseline G1 polyp measured 13 mm in diameter. Histology demonstrated a G1 tumour with a tiny focus of high-grade NEN (Ki67 40%). She subsequently continued endoscopic surveillance, with no further changes being observed to date (26 months).

The final patient demonstrated an increase in G1 NEN size from 20 to 30 mm after two surveillance endoscopies and 16 months follow up. Due to age and multiple co-morbidities, they were deemed unfit for a major resection and therefore underwent a gastric wedge resection instead. Histology demonstrated a well differentiated G1, 30 mm polyp and they remained under follow up for five years postoperatively with no further changes.

Two patients in this subgroup also developed additional dysplastic pathology in the stomach, distinct from their NEN during follow up. One patient who initially had multiple G1 NENs (largest 15 mm) developed an antral adenocarcinoma, 60 months after initial diagnosis and underwent a total gastrectomy. Histology showed a T1b N1 R0 gastric adenocarcinoma. This patient unfortunately died 15 months after the gastrectomy from a further different malignancy (cholangiocarcinoma). The second patient, who initially had a 12 mm G1 NEN was found to have a focus of gastric high-grade dysplasia 243 months after the original g-NEN diagnosis and underwent a successful endoscopic mucosal resection, with no evidence of further dysplasia being found 20 months post procedure.

### Patient Survival

**Fig. 2:**
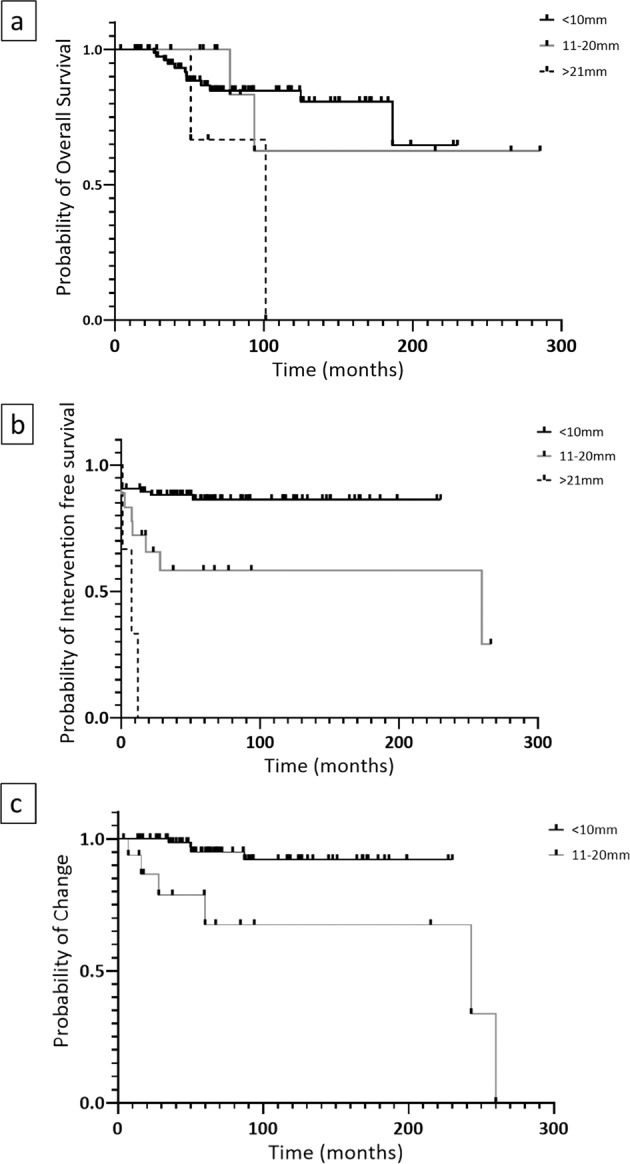
Effect of type I gastric NEN size on patient prognosis. Kaplan–Meier curve demonstrating probability of **a** survival depending on size, **b** Intervention free survival depending on size and c rate of change depending on size

No difference in overall survival (OS) was demonstrated between patients with tumours ≤10 mm and 11–20 mm. Patients with ≤10 mm tumours however survived significantly longer than those who had ≥21 mm tumours (OS undefined vs 101 months, *p* = 0.003) (Fig. 2a). Furthermore, patients with ≥21 mm tumours required further intervention with a median timeframe of 7.6 months (*p* < 0.001) from diagnosis, compared to patients with tumours 11–20 mm in whom further intervention was performed much later (median 260 months (*p* = 0.01)) (Fig. 2b). When examining the rate of change in either grade or size between patients with tumours <10 mm and 11–20 mm, a statistically significant difference was demonstrated, albeit this occurred over a prolonged period of time (Rate of change undefined vs 243 moths, *p* = 0.02) (Fig. 2c).

## Discussion

The management of type I g-NENs can be challenging. In our current study, we report the outcomes of 105 type I g-NEN patients who were managed at two ENETS Centres of Excellence in the UK. Patients had similar tumour characteristics to those described in previous cohorts, with a predominance of multiple, small, low grade g-NENs. 87 patients were enrolled into a ‘watch and wait’ endoscopic surveillance programme after no initial treatment and underwent regular endoscopic and histological surveillance with strict criteria defined for intervention. Of these 87 patients, only six (6.9%) were found to develop clinically meaningful changes in NEN size or grade during surveillance and in only two (2.3%) of these cases, did this lead to a change in patient management. Two additional patients in the endoscopic surveillance group developed gastric high-grade dysplasia or adenocarcinoma during follow up.

Currently, the only recognised risk factor for type I g-NEN progression is the size of tumour, with a cut-off for intervention being set by ENETS at 10 mm. Conservative management approaches involving endoscopic surveillance and/or endoscopic resection are therefore advocated in most patients. Some authors have proposed resection of all visible lesions, whilst others have set a size threshold for this [10, 11]. Endoscopic resection can be achieved by biopsy forceps, endoscopic mucosal resection (EMR), endoscopic submucosal dissection (ESD) and argon plasma coagulation (APC) [14–16], but there is currently no conclusive evidence of superior outcomes to support any individual technique over another [5].

Very few publications have investigated the role of active surveillance alone in type I g-NENs, but the available evidence suggests that patients who were managed in this way did not appear to progress after long periods of follow-up [17–20]. The largest cohort by Sato however included only 25 patients, but follow-up was for 204 months [19]. All included patients had favourable tumour characteristics of small size and low grade at baseline and no disease related deaths were noted in patients who were managed in this way. Two recent analyses of type I g-NEN surveillance programmes following endoscopic treatment [21, 22] have however suggested that approximately 30% of patients with type I g-NENs will require intervention in the form of endoscopic resection or surgery at some point during their disease course. A reintervention rate of at least 50% (7/13 patients and 44/84 patients in each study) was also noted after a median follow up of 22 and 11 months, respectively, after first intervention, with a further 50% of patients experiencing multiple recurrences. Local tumour recurrence rates ranged from 0–63.6%, depending on which series was reviewed (median follow up varied from 24 to 84 months) and appeared to be higher when simple polypectomy was performed using biopsy forceps or endoscopic snare [10].

It therefore remains unclear as to which patients with type 1 g-NEN are more suitable for endoscopic surveillance alone, how frequently this detects significant changes in NEN size/grade that require alterations in treatment and whether this management approach results in any adverse long term outcomes.

In our study, all patients undergoing endoscopic surveillance who had gastric NENs ≤10 mm at baseline did not demonstrate a clinically meaningful increase in tumour size during follow up, and although three patients developed minor alterations in tumour Ki67 index, none of these led to serious consequences or even a change in management plan. No serious complications occurred as a result of these surveillance procedures; however, repeated endoscopies can be unpleasant for patients and costly for the health care system. The indolent disease course of ≤10 mm type I g-NENs is reflected in the overall survival curves and mirrored in the intervention free survival curves. In view of this, the benefits of annual endoscopic surveillance in this group of patients should be questioned and if disease stability has initially been demonstrated, the intervals between endoscopic procedures could potentially be safely increased. Although we did not detect any cases of gastric adenocarcinoma in this group, intermittent endoscopic surveillance, maybe every 2–3 years, to detect this outcome is still probably appropriate in view of these patients’ increased susceptibility to developing this disease [23, 24].

The patients who were found to develop significant changes in their gastric pathology during endoscopic surveillance all had potentially more advanced tumours (≥11 mm in diameter) at baseline. These patients either chose a surveillance approach or were considered to be high risk for tumour resection at baseline. Five of the 12 patients in this category showed significant changes during follow up, including two who developed other gastric malignancies. As a result, we suggest that (in keeping with the current ENETS guidelines) initial tumour resection should probably be performed in most patients who have ≥11 mm type I g-NENs, if they are fit enough. However, endoscopic surveillance does appear to be a safe alternative strategy if resection is not possible for any reason.

None of the patients in our cohort who had an endoscopic NEN resection either initially or following a period of surveillance experienced a significant complication. Re-intervention was rare and lower than in other published series [21, 22], but our study employed more stringent criteria for defining a change in tumour status during follow-up up and tended to have a more conservative management strategy overall.

In our study, there was only one tumour-related death in the whole cohort. This patient had a very large and atypical 50 mm NEN and underwent a total gastrectomy. 24 of the remaining 104 patients have also died from other diseases, in keeping with the overall age profile of the cohort. No patients have developed distant metastases during follow up, and as far as we are aware (although most patients have not had routine surveillance cross sectional imaging), only one patient (as described in the Results section) has developed a local lymph node metastasis.

The strengths of the current study include comprehensive patient inclusion from prospective databases, endoscopic procedures being undertaken by the same small group of endoscopists, thus reducing interobserver variability, accurate patient characterisation at baseline and good follow up records. The study however has a number of limitations, including its retrospective nature, lack of Ki67 immunohistochemistry on the patients who were managed in the earlier part of the programme, the potential confounder that 8 patients also participated in a clinical trial of Netazepide for 12 months and lack of information about the cause of death in some patients. In addition it is possible that some patients in our region who had small type I g-NENs were managed locally in their district hospital rather than being referred to the ENETS Centre of Excellence.

In conclusion, our data support the established view that the majority of patients who have type I g-NENs have an excellent prognosis and are unlikely to develop distant metastases or die as a result of their disease. Initial endoscopic resection appears to be unnecessary in patients who have polyps ≤10 mm. Moreover, the recommendation that these patients should have surveillance endoscopies annually should potentially be reconsidered, as a longer interval between procedures is likely to be as safe, as well as less unpleasant and expensive. In our cohort, endoscopic resection was safe in patients who had g-NENs ≥11 mm and this intervention led to tumour resolution in most cases. However, if patients did not wish to have an endoscopic resection, a period of ‘watch and wait’ with a planned intervention only following an increase in tumour size or grade appeared to be a suitable alternative management option, that did not result in any long-term adverse outcomes. Periodic lifelong endoscopic surveillance should however still be undertaken in this group, as patients are still at risk of developing further NENs or gastric adenocarcinoma. The small minority of patients who have very unusual tumour characteristics at the time of presentation (e.g. large tumour size (>20 mm) or evidence of lymph node metastases) require a more aggressive initial surgical treatment plan and it is only this small subgroup of patients who appear to have a long-term risk of tumour-related death.

## Data Availability

Data available on request from the authors.
